# Idiopathic gastric fundus necrosis: Case report about a rare and fatal clinical condition

**DOI:** 10.1016/j.ijscr.2020.04.023

**Published:** 2020-05-08

**Authors:** Valeria Tognoni, Carlo Gazia, Anton Mariani Ivanikhin, Cristine Pathirannehalage Don, Dario Venditti

**Affiliations:** Department of Emergency Surgery, Policlinico Tor Vergata, “Tor Vergata” University Hospital, Viale Oxford 81, 00133 Rome, Italy

**Keywords:** AGD, acute gastric dilatation, POD, post-operative day, IGP, intra gastric pressure, Case report, Gastric fundus ischemia, Necrosis, Gastric surgery, Stomach, Acute gastric dilatation

## Abstract

**Introduction:**

Gastric fundus ischemia is a rare event, which does not account for many significant clinical studies. This disorder could have different etiologies, but authors agree that a prompt diagnosis and a proper treatment could avoid dangerous complications and ultimately the death of the patient.

**Presentation of case:**

We herein report an interesting idiopathic case of acute gastric dilatation and fundus ischemia of an 83-year-old Caucasic woman who was admitted to the Emergency Department complaining of abdominal discomfort, vomiting and constipation.

**Discussion:**

In literature, only a few case reports about this condition are reported. Possible risk factors, etiologies, and the different therapeutic options available for this condition are examined, in order to try to favor clinicians to formulate a timely diagnosis and provide patients with rapid healthcare services.

**Conclusion:**

Further investigations are still needed to analyze the pathophysiological pathways responsible of gastric fundus ischemia and to provide a definitive treatment to this dangerous disorder.

## Highlights

• Ischemic gastric necrosis could be provoked by acute gastric dilatation.
• Early diagnosis is crucial to increase survival from fundic gastric necrosis.
• Conservative treatments should be considered as first-line management.
• Surgical treatment is advised when massive gastric necrosis is encountered.

## 1 Introduction

One of the most frequent gastric diseases requiring surgery is represented by peptic ulcers, traumas and physical, chemical and iatrogen injuries. Ulcers are breaks affecting the inner lining of the stomach. They are usually spotted in the duodenum or in the stomach antrum or pylorus, while they are less diagnosed in the stomach fundus [1].

Only a few case reports are described in literature about acute necrosis and perforation of the gastric fundus, with different pathophysiological hypothesis. Gastric ischemic necrosis is characterized by inadequate blood supply to specific affected areas. There are several pathogenetic causes which may lead to a gastric necrosis, such as emphysematous gastritis [2], mucormycosis [3] and ischemic conditions related to acute gastric dilatation (AGD) [4,5]. In the present case report we analyzed the pathophysiological process which may be responsible for ischemia after AGD. This manuscript is reported in line with the SCARE criteria [6].

## 2 Presentation of case

**Fig. 1:**
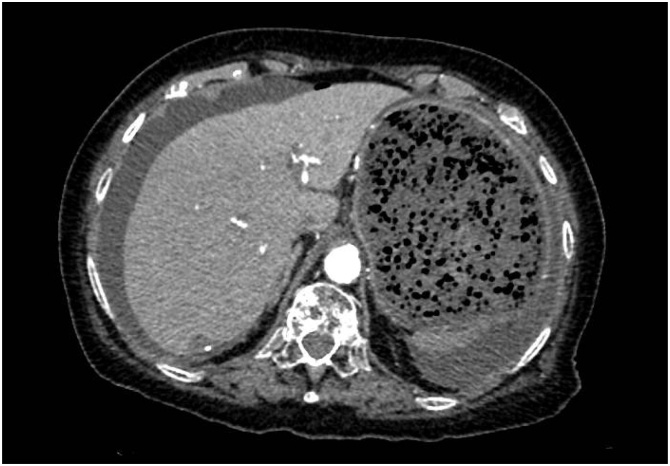
CT scan showing stomach dilatation.

**Fig. 2:**
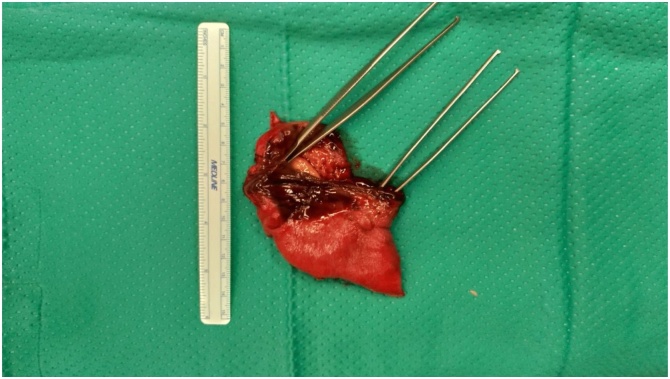
Necrotic specimen after first surgery.

An 83-year-old Caucasian woman with a history of hypertension, colon diverticulosis, hysterectomy and mastectomy after breast cancer was admitted to the Emergency Department. The patient complained of abdomen discomfort, vomiting and constipation for the last two days. After physical examination the abdomen was remarkable for tenderness with a positive Blumberg sign. Laboratory exams showed leukocytosis, with a progressive diuresis reduction. Abdominal CT scans demonstrated gastric distension and oesophageal dilatation, with free abdominal air and fluids (Fig. 1). An emergency laparotomy was performed after written informed consent was obtained from the patient. Multiple omental adhesions were found and more than 2.5 L of a brownish foul-smelling peritoneal liquid was drained. After exploring the bowel, a massive necrotic area of the anterior gastric fundus was found with a longitudinal perforation, which was close to the greater curvature. An atypical vertical gastric resection was performed Fig. 2.

On 3rd post-operative day (POD) a parenteral nutritional therapy was initiated and general conditions were satisfying overall. A chest CT described pleural effusion, with no further alterations. However, the patient’s clinical conditions did not allow the execution of a gastrografin study. Therefore, a methylene blue oral test was performed, which resulted in no gastric leak. Hence, the nasogastric tube was removed and a hydric diet was well-tolerated.

Unexpectedly, on 7th POD, a bile-like green liquid was seen both from the surgical incision and the surgical wound drains.

An emergency re-laparotomy revealed a biliary peritonitis with minimal different perforation of the residual gastric fundus, next to the esophagogastric junction. Therefore, a formal sleeve gastrectomy was performed.

After the second surgery, on 3rd POD, the nasogastric tube was accidentally removed by the patient and it was not repositioned due to the respiratory distress of the patient. Clinical findings showed a progressive lung function loss, while a routine arterial blood gas exam documented a metabolic alkalosis. High blood pressure, tachycardia, fever, oliguria and a surgical wound infection were documented. After nephrological counseling, plasma and high-dosage furosemide were prescribed to sustain the kidney function. Vancomycin and meropenem were administrated to prevent sepsis. On 4th POD lactates were found to be elevated and the echocardiographic ejection fraction was 35 %, with a biliary leakage from the abdominal drains and the surgical wound. A 3rd laparotomy was then performed. A biliary peritonitis with a surgical dehiscence was confirmed close to the gastric antrum. A gastrorraphy of the surgical leak was performed and reinforced with fibrin glue. The peritoneal cavity was abundantly debrided and irrigated with isotonic saline solution.

The postoperative management was affected by a severe cardiorespiratory failure and a hydroelectrolytic discrepancy. After five days, the surgical drains and the wound showed a bile-leak yet again. Laboratory findings documented bile in the surgical drains. The patient died on 8th POD.

After histopathological examination of both specimens, a gastric transmural ischemic infarction with serositis was documented.

## 3 Discussion

Nowadays, gastric necrosis of the fundus is a rare condition. The actual incidence is unknown. In literature, less than 50 cases of AGD with ischemia and necrosis are reported [7]. This condition usually carries a poor prognosis with no univocal interpretation. Different authors documented cases connected to venous insufficiency due to AGD, while others are related to mucormycosis, which is an angioinvasive fungal infection, as a possible etiology. Primary gastric mucormycosis might be caused by inoculation or by a fungal infection of the stomach, which causes infection and then leads to a gastric necrosis. Secondary mucormycosis may appear after fungal dissemination in the bloodstream [8]. Infections can occur with pathological and microbiological findings of necrotic gangrene and microorganisms.

Emphysematous gastritis is an uncommon infection of the gastric wall usually caused by *Streptococci*, *E. coli*, *Enterobacter* spp., *S. Aureus* and *Clostridium*. CT scans are diagnostic because of the characteristic presence of gas in the gastric wall and stomach distention. Several factors might affect the integrity of the gastric mucosa, while others favor infections. Gastric infections, ingestion of corrosive substances, recent surgery, diabetes, rheumatic diseases and corticosteroids are included. Fulminant infections present with the classic pathognomonic sign of necrotic nasogastric aspirate. When a perforation is found, a surgical treatment is paramount [9].

AGD is defined when the intra gastric pressure (IGP) from distention is above 30cmH2O. Then, IGP begins to impair the intramural blood flow, resulting in ischemia and necrosis of the stomach. However, besides eating disorders, other etiologies of this condition are undetermined [10].

To the best of our knowledge, a gastric ischemic necrosis, which is not correlated with all of the abovementioned conditions, is an extremely rare event. Primitive gastric ischemia, either acute or chronic could be usually ascribed to systemic hypotension, gastric volvulus, disseminated thromboembolism, vasculitis or atherosclerosis. Gastric ulcers of the fundus are sporadically diagnosed, but when it presents as a chronic ischemia it is not advised to treat it with protonic pump inhibitors.

In the current case report short gastric arteries and gastric arteries were completely pervious, so the most plausible pathogenesis seems related to a venous infarction. The hypothesis of gastric necrosis due to bacterial or fungal infections was declined by negative microbiological cultures. The idiopathic gastric necrosis of the fundus was described as a consequence of the acute increase in intragastric pressure. This event could, at first, reduce the local blood flow to the mucosa and subsequently to the general venous flow [11].

Multiple reports were attempted to describe the etiology and pathogenesis of AGD. The simplest explanation and relatively frequent cause of AGD is extrinsic or intrinsic obstruction of the gastrointestinal tract. Nonetheless, in literature there are also multiple interesting correlations of AGD with different diseases such as muscular dystrophy [12] or diabetes [13].

In our case, there was no evidence of all the aforementioned causes of extrinsic or intrinsic obstruction of the gastrointestinal tract, and a similar case was already described by Todd et al. [14]. Despite the gastric dilatation of that patient not being as big as the one that our group described, it still seems that there was a strong connection with a general venous flow congestion.

The second gastric perforation we described states the same histological conclusion of ischemic necrosis. This finding, when all other possible etiologies were discarded, suggests a plausible situation of idiopathic gastric paralysis with consequent AGD.

A fast response is mandatory when AGD is encountered, since it might quickly become a life-threatening entity. Delaying the diagnosis process of AGD is proven to be fatal in more than 80 % of cases [15].

According to our group, the surgical approach must be inspired by two general rules: 1) damage control, to stabilize the clinical situation of the patient; 2) sparing surgery, avoiding invasive gastric surgical operations every time it is indicated, saving as much healthy tissue as possible, in contrast with other authors [16]. Since the etiopathological causes are not well-defined, we prefer this conservative surgical approach. According to Steen et al., a total gastrectomy could be the safest option [16], but it is also the most invasive treatment, and conservative managements represent a valid option if early instituted. Patients undergoing a partial gastric resection experience a better quality of life when discharged, since part of the stomach is still preserved [17]. For this purpose, we prefer to perform partial gastric resections or, when clinical conditions are not favorable, our approach relies on gastric decompression to avoid biliary leaks. In similar conditions, a Petzer catheter or a Kehr tube surrounded by gastric omentum could be positioned to create a gastrostomy that needs an uncomplicated future reversal surgery. The extreme damage and the great amount of unhealthy gastric tissue encountered in our patient did not allow the execution of a gastrostomy decompressive treatment. In the end, a gastroesophageal stenting as a primary measure after leakage is worthwhile in emergency cases only after positioning a surgical or radiological drainage, when the leakage is small-sized and confined to the paraoesophageal area [18]. Some reports debate if a partial or total gastrectomy should be performed with or without feeding jejunostomy to provide an adequate caloric input [19].

## 4 Conclusion

Several reports about acute fundus gastric necrosis are missing a definitive etiology, despite all of them containing risks. Different hypothesis were advanced, but a deeper knowledge of the disease is required to fully determine the best therapeutic strategy.

## Declaration of Competing Interest

None.

## Sources of funding

None.

## Ethical approval

Ethical approval has been exempted by our institution.

## Consent

Written informed consent was obtained from the patient for publication of this case report and accompanying images. A copy of the written consent is available for review by the Editor-in-Chief of this journal on request.

## Author contribution

Valeria Tognoni: conception and design of the study, analysis of data, manuscript revision, final manuscript approval; Carlo Gazia: conception and design of the study, analysis of data, manuscript drafting, manuscript revision, final manuscript approval; Anton Mariani Ivanikhin: conception and design of the study, manuscript drafting, manuscript revision, final manuscript approval; Maurizio Rho: manuscript revision, final manuscript approval; Andrea Martina Guida: manuscript revision, final manuscript approval; Cristine Pathirannehalage Don: manuscript revision, final manuscript approval, Dario Venditti: conception and design of the study, analysis of data, manuscript revision, final manuscript approval.

## Registration of research studies

No registration for this case report is needed.

## Guarantor

Dario Venditti.

## Provenance and peer review

Not commissioned, externally peer-reviewed.
